# Analysis of clinical factors and inflammatory cytokines in patients with lung cancer and sarcopenia: a prospective single-center cohort study

**DOI:** 10.3389/fonc.2025.1564399

**Published:** 2025-04-08

**Authors:** Yalan Liu, Hui Zhang, Peng Chen, Xinfu Liu

**Affiliations:** ^1^ Department of Oncology, The Central Hospital of Shaoyang, Shaoyang, China; ^2^ Department of Thoracic Oncology, Tianjin Medical University Cancer Institute & Hospital, National Clinical Research Center for Cancer, Tianjin Key Laboratory of Cancer Prevention and Therapy, Tianjin’s Clinical Research Center for Cancer, Tianjin, China

**Keywords:** lung cancer, sarcopenia, cytokines, malnutrition, prognosis

## Abstract

**Objective:**

To analyze the relationship between the expression of various clinical factors, inflammatory cytokines, and sarcopenia and provide new ideas for whole-course management and curative effect prediction in patients with lung cancer and sarcopenia.

**Methods:**

A total of 135 patients with lung cancer recruited in the Department of Oncology, Central Hospital of Shaoyang, from January 2022 to January 2024 were analyzed and divided into sarcopenia (75 cases) and non-sarcopenia (60 cases) groups. Various statistical analyses methods were used to analyze the correlation between 4 kinds of inflammatory cytokines and sarcopenia in patients with lung cancer.

**Results:**

In this study, 55.6% (75/135) of the lung cancer patients were found to have sarcopenia, with a median age of 67.3 years. Those with sarcopenia were found to be significantly associated with increased age, long duration of cigarette inhalation, and high risk of malnutrition. The results of the regression analysis indicated that long-term cigarette inhalation (odds ratio [OR]=8.187), body mass index (BMI; OR=1.356), and Nutritional Risk Screening 2002 score (OR=0.050) were statistically significant (*P*<0.05). Multivariable logistic regression analysis indicated that patients in the sarcopenia group were positively correlated with interleukin (IL)-6 and tumour necrosis factor (TNF)-α (*P*<0.05). The progression-free and overall survival of lung cancer patients with sarcopenia who received chemotherapy were significantly increased compared to those who did not receive chemotherapy(*P*<0.05).

**Conclusions:**

Patients with a long-term cigarette inhalation, high risk of malnutrition, and low BMI have a higher probability of sarcopenia. The increased expression levels of IL-6 are positively correlated with the occurrence of sarcopenia, as well as TNF-α. The intervention of chemotherapy affects inflammatory cytokine levels. Early chemotherapy may extend the survival time of patients with lung cancer and sarcopenia.

## Introduction

Patients with advanced-stage lung cancer often have nutritional and metabolic disorders. Compared with that in patients suffering from other solid tumors, the risk of nutritional and metabolic disorders and cachexia in patients with lung cancer is higher, at approximately 47.1%–56.2% (1). Patients with lung cancer and sarcopenia tend to have worse prognosis and overall survival that those without sarcopenia. With the occurrence and development of tumors, the competition for nutrients between tumor tissues and patients leads to the consumption of large amount of nutrients, such as lipids, amino acids, and glucose in the body. The muscle protein synthesized by the body is gradually reduced, while the catabolism speed is gradually increased, making patients with malignant tumors more susceptible to sarcopenia.

The concept of sarcopenia was earliest proposed by Professor Rosenberg in 1989 (2) and is now widely used in clinical practice. According to European Working Group on Sarcopenia in Older People (EWGSOP) in 2018 (3), the European consensus updated the definition of sarcopenia as a clinical state of muscle atrophy with reduced function that can lead to adverse consequences including reduced qualify of life (4). Due to the different body sizes and genetic backgrounds of Asian populations, the Asian Working Group for Sarcopenia (AWGS) proposed a consensus on the disease in 2014 and updated it in 2022 (5). As reported previously, changes of various inflammatory and immune factors in patients with advanced-stage lung cancer are related to their prognosis to some extent (6). With increasing age, the expression levels of cytokines in older patients changes, especially in malignant tumors, which increase the expression levels of pro-inflammatory factors. Meanwhile, aging also decrease the levels of anti-inflammatory factors in the body. This situation will ultimately give rise to a chronic inflammatory state with low levels of cytokines, which can also be called immune aging (7). A correlation exists between tumor microenvironment and the clinical prognosis of patients with advanced-stage lung cancer (8). However, the correlation between the inflammatory cytokines in the special physiological environment of advanced-stage lung cancer and sarcopenia still requires clinical validation.

For this study, we collected and analyzed the correlation between clinical data and levels of serum inflammatory cytokines (interleukin [IL]-2, IL-6, IL-8, and tumor necrosis factor [TNF]-α) to guide the prognosis of patients who suffered advanced-stage lung cancer.

## Materials and methods

### Study design and patients

A total of 135 patients with lung cancer recruited to the Department of Oncology at the Central Hospital of Shaoyang between January 2022 and January 2024 were studied. The inclusion criteria are as follows: 1) age 40–80 years old; 2) inoperable or metastatic lung cancer confirmed via pathological biopsy in the outpatient or oncology ward of our hospital with measurable tumor lesions; 3) Eastern Cooperative Oncology Group (ECOG) Performance Status score (PS) <2 points; and 4) completion of a body composition test and providing the basic information according to the research requirements. The exclusion criteria are as follows: 1) complications with multiple organ dysfunction, immunodeficiency disease, or digestive dysfunction complicated by a second tumor other than lung cancer; 2) neurological disorders, mental disorders, and inability to communicate normally, with a long history of alcohol or drug abuse; and 3) a history of open surgery or trauma within the last 3 months. Before participating in the clinical study, all patients were informed of the precautions in detail and provided their signed informed consents. This clinical study was approved by the Ethics Committee of Shaoyang Central Hospital (KY2023-002-29) at the time of the project establishment. All procedures involving human participants were performed in accordance with the ethical guidelines outlined in the Declaration of Helsinki.

### Data collection

The history of present disease of recruited patients was reviewed, and the age, sex, smoking history and clinical stage of the patients were documented. The pathology query system was utilized to record pathology subtype of lung cancer diagnosis. Clinical data such as chemotherapy contraindications, chemotherapy regimens, progression-free survival (PFS), and overall survival (OS) of first-line treatment were recorded during follow-up. Fasting venous blood samples were collected according to the study requirements to measure the patients’ serum albumin, triglyceride, lymphocyte ratios, and the baseline levels of 4 kinds of cytokines (IL-2, IL-6, IL-8, and TNF-α).The expression levels of these cytokines were simultaneously determined via a technology called Cytometric Bead Array (CBA). Long-term cigarette inhalation was defined as smoking ≥10 cigarettes/day, continuous regular smoking ≥10 years. BMI was calculated as body mass/height^2^ (kg/m^2^).

Combined with the diagnostic requirements developed by the AWGS (9), muscle atrophy or decline in quality and quantity was classified as sarcopenia. The patients participated in the study were divided into two groups (with/without sarcopenia).

The human body composition analyzer uses current to calculate the composition of the human body through its resistance of the human body, which is a noninvasive, safe, accurate, and economical detection method for patients with advanced physical conditions. In the fasting state, the patient was asked to remove jewelry, coat, shoes, and socks, and stand upright on an InBody 970 body composition analyzer plate (Biospace China Corp. Ltd., Shanghai, China). The heel and sole planes of the foot should be close to the foot electrode, the upper limb should be placed naturally on the side of the body, and the hands should contact the hand electrode simultaneously for testing. The muscle mass data of the extremities were analyzed using a computational system: skeletal muscle mass index (SMI) = limb muscle mass/height^2^ (kg/m^2^), defined as reduced SMI <7.0 kg/m^2^ for men and <5.7 kg/m^2^ for women.

A Wanqing WCS-10 000 electronic grip dynamometer (Huayue Sports Goods Corp. Ltd., Yiwu, China) was employed to measure grip strength. The recruited patients should maintain their bodies upright during the inspection. The dominant hand was used to hold the grip dynamometer handle with maximum strength, and the feet were naturally separated. If the grip strength was <26 kg for men and <18 kg for women, we defined it as a decrease in muscle strength.

In a quiet environment, the time required for walking 6 m at the patients’ usual pace was recorded and accurately measured using a stopwatch. If the patient’s walking distance was less than 0.8 m/s, it was defined as reduced activity ability.

Nutritional Risk Screening 2002 (NRS2002) (10) was used to evaluate the nutritional status of the patients. The NRS2002 includes the disease score, age, and nutritional status. The total score for each indicator is not more than 5 points. A total score of at least 3 was considered to indicate a significantly increased risk of malnutrition. For patients at a high risk of malnutrition, nutrition physicians were asked to guide nutritional intervention therapy (such as balanced nutrition education, enteral nutrition solution, and intravenous nutrition solution), and the NRS2002 score of the patients was recorded after treatment.

For the 2 groups of patients with lung cancer, the expression levels of inflammatory cytokines at enrollment were measured as baseline data. For patients receiving first-line chemotherapy, the levels of included cytokines were measured again after the 2^nd^ and 4^th^ cycle of chemotherapy to evaluate the therapeutic efficacy. Based on the evaluation of head, chest, and abdomen enhanced computed tomography after 2 cycles of chemotherapy, the patients were categorized according to Response Evaluation Criteria in Solid Tumors (RECIST 1.1): complete response (CR), partial response (PR), progressive disease (PD), and stable disease (SD); the tumor control rate was calculated as CR + PR + SD (11).

The PS scoring of participated patients using the Zubrod-ECOG-World Health Organization (ZPS) 5 scores can be interpreted as follows: score 0: the activity levels are completely normal and that there is no difference between the activity levels before and after the onset of disease; score 1: mild symptoms, engaging in mild physical activity, including daily household or simple desk work; score 2: self-care but has lost the ability to work, daytime bedtime does not exceed 50%; score 3: only partially self-care, over 50% of the time in bed or in a wheelchair during the day; score 4: bedridden, cannot take care of themselves; and score 5 points: death.

### Outcome measures and statistical analysis

Statistical analyses were performed using SPSS software (version 27.0; IBM Corp., Armonk, NY, USA). The statistical data are expressed using absolute value, and the χ2 test was used to compare the data of the 2 groups. Measurements conforming to the normal distribution are expressed as ( $\bar{x}$ ± s). OS was defined as the time from the start of randomization to death or last follow-up, whereas PFS was defined as the time interval between the start of treatment and observation of disease progression or last follow-up. The final follow-up was conducted on December 25, 2024. OS and PFS were generated using Kaplan–Meier curves. An independent sample t-test was used for comparisons between groups. Univariate, binary logistic regression, and Pearson’s correlation analyses were used to determine the correlation between risk factors and cytokines that may be associated with lung cancer complicated by sarcopenia. Factors with *P*<0.15 were set as relevant factors, and *P*<0.05 as statistically significant differences.

## Results

### Baseline data

A total of 104 male patients and 31 female patients were included in our study. The mean age of all the patients was (63.2 ± 8.9) years. The sarcopenia and without-sarcopenia groups comprised of 75 (55.6%) and 60 (44.4%) patients, respectively. The sarcopenia group consisted of 60 patients with non-small cell lung cancer (NSCLC) and 15 patients with small cell lung cancer (SCLC). The without-sarcopenia group consisted of 51 patients with NSCLC and 9 patients with SCLC. The sarcopenia group had significantly higher age, long-term cigarette inhalation history, and nutritional screening scores than those without ones. The ratios of BMI, limb muscle mass, and SMI in the sarcopenia group were significantly decreased, and the differences were statistically significant (*P*<0.05). However, there were no statistically significant differences in sex, pathological biopsy type, clinical stage, PS score, serum albumin level, lymphocyte proportion, or triglyceride level between the two (*P*>0.05; **Table 1**).

**Table 1:** Comparison of included factors between the two groups of patients with lung cancer.

Inclusion factors	Sarcopenia group (n=75)	Non-sarcopenia group (n=60)	t/χ2	P
Age ( $\bar{x}$ ± s, years)	67.3 ± 7.3	58.1 ± 8.0	7.045	<0.001
Sex			0.253	0.615
Male	59 (78.7)	45 (75.0)		
Female	16 (21.3)	15 (25.0)		
Number of long-term smoking cases	55 (73. 3)	23 (38.3)	16.738	<0.001
Pathological type			0.570	0.450
NSCLC	60 (80.0)	51 (85.0)		
SCLC	15 (20.0)	9 (15.0)		
Clinical stage			0.065	0.799
III	21 (28.0)	18 (30.0)		
IV	54 (72.0)	42 (70.0)		
PS score (points)			0.285	0.594
0	23 (30.7)	21 (35.0)		
1	52 (69.3)	39 (65.0)		
BMI ( $\bar{x}$ ± s, kg/m^2^)	21.42 ± 2.07	23.13 ± 1.75	-5.119	<0.001
Muscle mass ( $\bar{x}$ ± s, kg)	16.14 ± 1.31	20.03 ± 1.73	-14.842	<0.001
SMI ( $\bar{x}$ ± s, kg/m^2^)	5.87 ± 0.54	6.67 ± 0.43	-9.35	<0.001
Serum albumin levels ( $\bar{x}$ ± s, g/L)	36.92 ± 3.11	37.49 ± 2.61	-1.130	0.260
Lymphocyte ratio ( $\bar{x}$ ± s)	0.37 ± 0.10	0.34 ± 0.08	1.899	0.060
Triglyceride levels ( $\bar{x}$ ± s, mmol/l)	1.19 ± 0.43	1.31 ± 0.47	-1.439	0.152
NRS2002 score ( $\bar{x}$ ± s, points)	3.77 ± 1.12	1.67 ± 0.97	11.511	<0.001

PS, physical status; BMI, body mass index; SMI, skeletal muscle mass index; NRS2002, Nutritional Risk Screening 2002 Scale.

### Binary logistic regression analysis of influencing factors of the sarcopenia group

Considering sarcopenia as the dependent variable (no assignment: 1, yes assignment: 2), the factors associated with sarcopenia were: age (assignment was the measured value), sex (male assignment was 1, female assignment was 2), whether there is a long-term cigarette inhalation (no assignment was 1, yes assignment was 2), BMI (assignment was the measured value), NRS2002 score (assignment: measured value), serum albumin level (assignment: measured value), lymphocyte ratio (assignment: measured value), and triglyceride level (assignment: measured value) were the independent variables. According to binary logistic regression analysis, we could find that long-term cigarette inhalation, BMI value, and nutritional screening score were the influencing factors of the sarcopenia group (*P*<0.05). However, age, sex, serum albumin, lymphocyte ratio, and triglyceride level could not be used as influencing factors for lung cancer complicated by sarcopenia, and there was no statistical significance (*P*>0.05; **Table 2**).

**Table 2:** Binary logistic regression analysis on influencing factors of lung cancer combined with sarcopenia.

Variable	β	SE	Wald χ2	P	Odds ratio (95% confidence interval)
Age	0.076	0.047	2.618	0.106	0.927 (0.846, 1.016)
Sex	0.772	0.956	0.651	0.420	0.462 (0.071, 3.012)
Long history of smoking	2.103	0.772	7.425	0.006	8.187 (1.804, 37.144)
Body mass index	0.304	0.161	3.567	0.047	1.356 (0.989, 1.859)
Nutritional Risk Screening 2002 score	2.829	0.632	20.046	<0.001	0.050 (0.017, 0.204)
Serum albumin levels	0.027	0.127	0.044	0.834	1.027 (0.801, 1.317)
Lymphocyte ratio	7.171	4.262	2.831	0.092	0.001 (0.000, 3.261)
Triglyceride levels	1.082	0.789	1.877	0.171	2.949 (0.628, 13.855)

### Comparison of peripheral blood inflammatory cytokines between the groups

The expression levels of peripheral blood cytokines from 135 patients with lung cancer (both sarcopenia and without sarcopenia group) were collected for statistical analysis at the time of enlistment. The results indicated no statistically significant difference in baseline levels of IL-2 and IL-8 between the 2 groups (*P*>0.05). However, the expression levels of IL-6 were statistically significant between the two groups (*P*<0.05), as well as TNF-α. Therefore, the occurrence of sarcopenia may have a certain impact on the expression levels of the 2 kinds of cytokines in the patients’ peripheral blood as shown in **Table 3**.

**Table 3:** Comparison of serum levels of inflammatory cytokines in the two groups of patients with lung cancer.

Cytokine species ( $\bar{x}$ ± s, pg/mL)	Sarcopenia group (n=75)	Non-sarcopenia group (n=60)	t	P
IL-2 level	10.17 ± 9.82	9.98 ± 7.48	0.122	0.903
IL-6 level	14.09 ± 4.87	5.84 ± 3.33	4.215	<0.001
IL-8 level	13.22 ± 6.61	12.84 ± 6.37	0.334	0.739
TNF-α level	16.36 ± 6.84	8.87 ± 3.93	7.983	<0.001

IL, interleukin; TNF, tumor necrosis factor.

### Multivariable logistic regression analysis of lung cancer with sarcopenia and inflammatory cytokines

To adjust for potential confounders, a binary logistic regression model was applied to compare cytokine levels between the two groups. The dependent variable (sarcopenia) was a binary classification (0= absent, 1= present), and continuous independent variables included levels of four inflammatory cytokines: IL-2, IL-6, IL-8, and TNF-α. After adjusting for age, smoking history, BMI, nutritional score, and disease stage, the results demonstrated that elevated IL-6 levels were positively associated with sarcopenia risk (OR=1.229, 95% CI: 1.082–1.399, *P*=0.025). Similarly, higher TNF-α levels independently correlated with increased sarcopenia risk (OR=1.380, 95% CI: 1.073–1.755, *P*=0.012), indicating that both IL-6 and TNF-α were independently linked to sarcopenia. No significant associations were observed for IL-2 or IL-8 (*P* > 0.05) as shown in **Table 4**.

**Table 4:** Multivariable logistic regression analysis between sarcopenia and inflammatory cytokines in patients with lung cancer.

Variable	β	SE	Wald χ2	P	Odds ratio (95% confidence interval)
IL-2	-0.002	0.075	0.001	0.975	0.998 (0.862, 1.155)
IL-6	0.028	0.078	1.131	0.025	1.229(1.082, 1.399)
IL-8	-0.108	0.097	1.255	0.263	0.897(0.743, 1.085)
TNF-α	0.322	0.129	6.277	0.012	1.380(1.073, 1.755)
Age	-0.084	0.057	3.524	0.103	0.827(0.724, 1.028)
Long history of smoking	3.286	0.792	8.369	0.119	2.187(1.804, 3.194)
Body mass index	0.304	0.161	3.567	0.087	1.356(0.989, 1.859)
Nutritional Risk Screening 2002 score	-2.789	0.789	5.046	1.124	0.089(0.027, 0.186)
Clinical stage	1.059	0.675	1.897	0.171	2.849(1.928, 3.855)

IL, interleukin; TNF, tumor necrosis factor.

### Comparison and efficacy evaluation of IL-6 and TNF-a levels before and after chemotherapy in the two groups

After clinical evaluation, exclusion of contraindications, and obtaining informed consents, 40 patients from the sarcopenia group and 45 from the without-sarcopenia group administered chemotherapy. The expression levels of the 2 kinds of cytokines in patients’ peripheral blood were measured after 2^nd^ and 4^th^ cycle of chemotherapy, and the therapeutic effect was evaluated using RECIST 1.1. There was a statistically significant difference in the evaluation of efficacy after chemotherapy between the 2 groups (*P*<0.05). There was also a statistically significant difference in IL-6 levels between the 2 groups before chemotherapy (*P*<0.05); however, there was no statistically significant difference observed after chemotherapy (*P*>0.05). Similar results were obtained for the difference in TNF-a levels between the 2 groups, as shown in **Table 5**.

**Table 5:** Comparison and efficacy evaluation of IL-6 and TNF-a levels before and after chemotherapy in the two groups of patients with lung cancer.

The level of cytokine ( $\bar{x}$ ± s, pg/mL)	Sarcopenia group (n=40)	Non-sarcopenia group (n=45)	t/χ2	P
IL-6 level				
Prechemotherapy	16.74 ± 15.18	5.87 ± 3.63	4.657	<0.001
After 2 cycles	10.62 ± 5.67	12.24 ± 4.34	1.492	0.139
After 4 cycles	10.05 ± 3.28	14.38 ± 5.72	2.152	0.247
TNF-α level				
Prechemotherapy	15.82 ± 7.63	9.39 ± 3.83	4.810	<0.001
After 2 cycles	11.85 ± 4.79	10.95 ± 6.56	0.730	0.467
After 4 cycles	9.65 ± 1.26	12.28 ± 2.47	3.724	0.629
After 2 cycles			6.649	0.010
PR + CR + SD (n%)	25 (62.5)	39 (86.7)		
PD (n%)	15 (37.5)	6 (13.3)		
After 4 cycles			8.379	0.004
PR + CR + SD (n%)	19 (47.5)	35 (77.8)		
PD (n%)	21 (52.5)	10 (22.2)		

IL, interleukin; TNF, tumor necrosis factor; PR, partial response; CR, complete response; SD, stable disease; PR + CR + SD (effective treatment); PD, disease progression (ineffective treatment).

### Survival analysis of patients with lung cancer and sarcopenia

**Figure 1:**
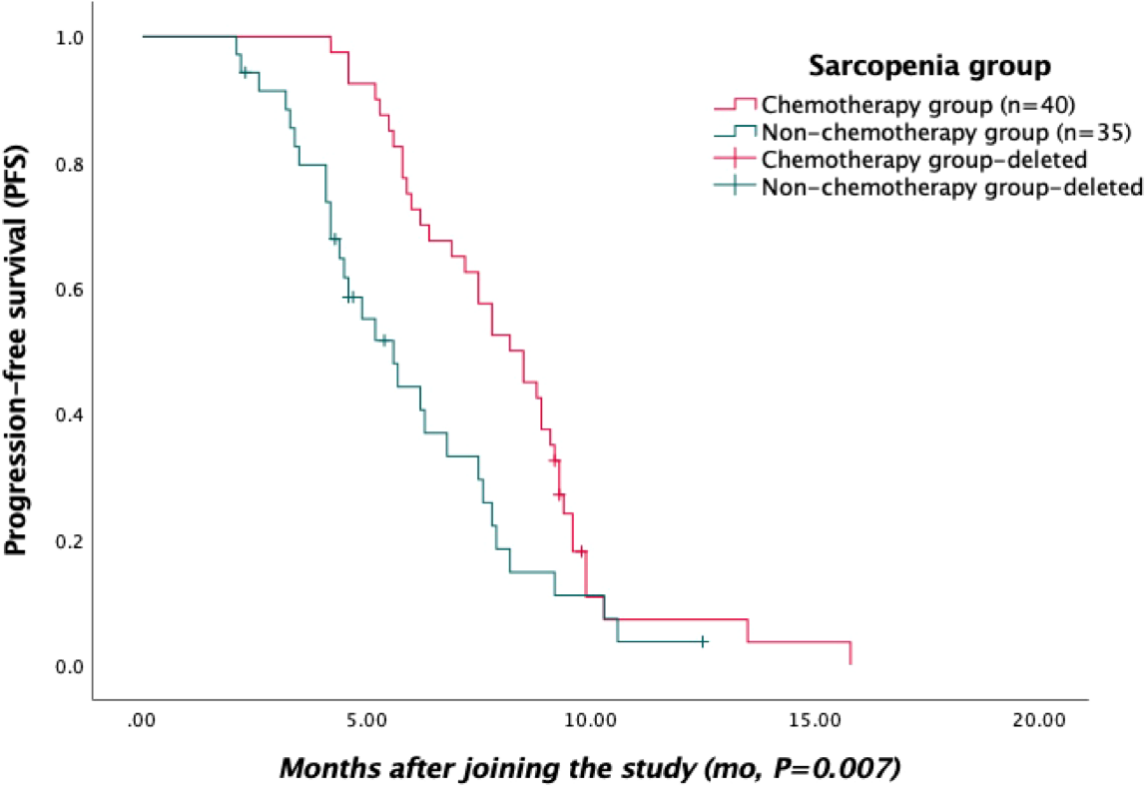
Kaplan–Meier curves of progression-free survival (PFS) in the sarcopenia group.

**Figure 2:**
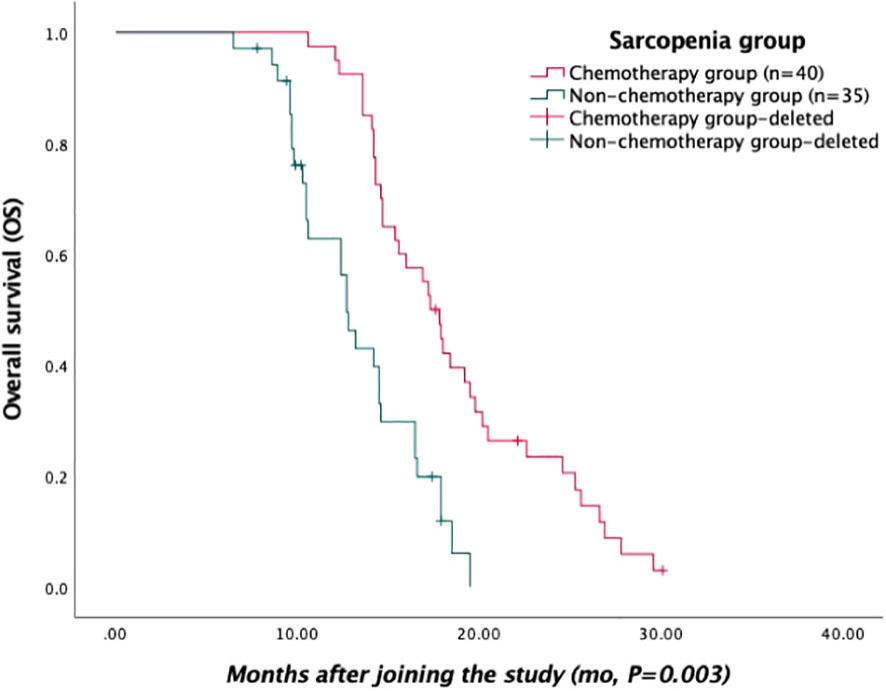
Kaplan–Meier curves of overall survival (OS) in the sarcopenia group.

Survival data, including PFS and OS after first-line treatment, were recorded in 75 patients from the sarcopenia group through medical records and telephone follow-ups. Five patients were lost during follow-up. Kaplan–Meier curves were used to generate PFS and OS (**Figures 1**, **2**, respectively). According to the survival curves, the PFS and OS in the chemotherapy group were higher than those in the non-chemotherapy group (*P*<0.05).

### Analysis of sarcopenia group after nutritional treatment intervention

**Figure 3:**
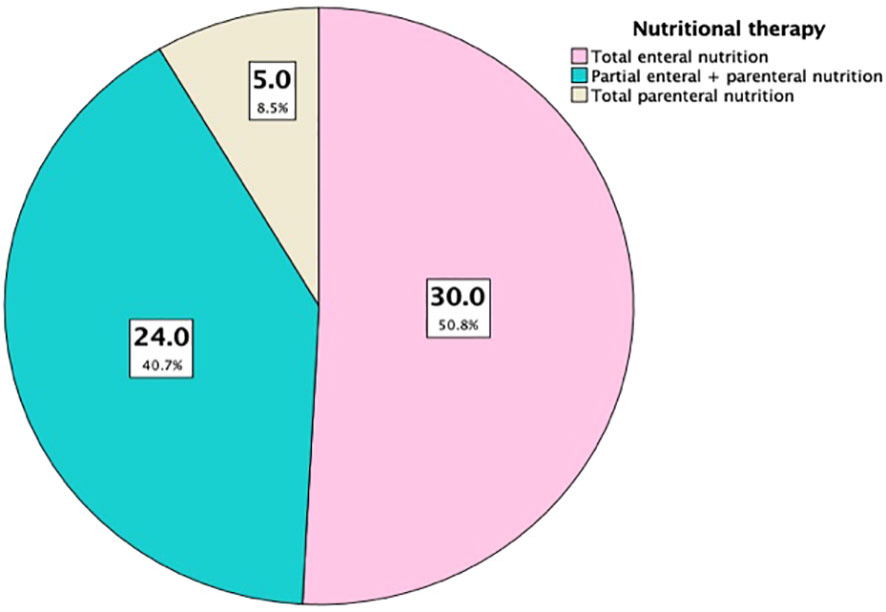
Different nutritional treatments for the sarcopenia group (n=59).

We screened and assessed the nutritional risk of 75 patients with lung cancer and sarcopenia, and found that 59 patients (78.7%) of them were at high risk of malnutrition. After fully assessing the physical condition of the patients, we asked the nutritionist of our research center to consult and formulate an appropriate nutritional treatment plan for these 59 patients. According to the records of medical system, 30 of them were supplemented with enteral nutrient solution under the premise of daily diet. In 24 patients, part of the enteral nutrition solution and parenteral intravenous nutrition were mutually complementary. Total parenteral nutrition (TPN) was performed in 5 patients who were unable to obtain sufficient energy by oral means (**Figure 3**). After 1 month of treatment, we collected clinical data and cytokine level results of these 59 patients for statistical analysis. Although the BMI and SMI values of 59 patients had a certain upward trend after nutritional treatment intervention, there was no statistically significant difference (*P* > 0.05). The expression levels of IL-6 and TNF-a in peripheral blood of patients with different nutritional treatments in the 3 groups were decreased to a certain extent after treatment, but there was no statistically significant difference (*P* > 0.05) (**Table 6**).

**Table 6:** Comparison of different clinical factors before and after nutritional treatment (n=59).

Comparison items ( $\bar{x}$ ± s)	Pre-nutritional therapy	Post-nutritional therapy	t	P
BMI (kg/m^2^)	20.17 ± 1.76	20.65 ± 1.83	-1.44	0.154
SMI (kg/m^2^)	6.05 ± 0.26	6.12 ± 0.27	-1.31	0.191
IL-6(pg/ml)	15.91 ± 2.37	15.65 ± 1.38	0.70	0.488
TNF-α(pg/ml)	23.55 ± 3.07	22.34 ± 3.93	1.86	0.066

BMI, body mass index; SMI, skeletal muscle mass index.

## Discussion

Sarcopenia was originally considered a disease affecting older individuals; however, it is not limited to them and can occur alongside many diseases (12). It can increase the incidence of falls, pathological fractures, dysfunction, hospitalization, and death, leading to a significant medical burden (13). Some researchers believe that sarcopenia is a degenerative disease accompanied by decreased skeletal muscle content and activity with age (14). With continuous clinical exploration, it has been found that in addition to the age-related occurrence of sarcopenia, patients with other wasting diseases at baseline or severe malnutrition caused by a second tumor may be prone to an increased incidence of sarcopenia (15). A previous study mentioned that the incidence of lung cancer combined with sarcopenia was approximately 47.1%–56.2% (16), which was consistent with the incidence of sarcopenia (55.6%) obtained in the current study. Regression analysis of independent risk factors for lung cancer with sarcopenia revealed that long-term cigarette inhalation, lower BMI, and higher risk of malnutrition were more clinically significant outcomes. Through the analysis of peripheral blood inflammatory cytokines, it was found that lung cancer complicated with sarcopenia may have a certain effect on the expression levels of proinflammatory cytokines in peripheral blood. After the intervention of chemotherapy, the expression levels of these cytokines intergroup tended to be similar. This finding suggests that chemotherapy may alter the cellular immune status of patients with lung cancer, as also reported previously (17). We followed up patients with lung cancer complicated with sarcopenia for up to 3 years and found that patients who administered first-line chemotherapy had significant benefits in survival time compared with those who did not administer chemotherapy, suggesting that early intervention in terms of clinical treatment should be taken to maximize their quality of life and prolong their survival time. Due to the short observation period of this study, patients with a high risk of malnutrition still had a certain degree of nutritional risk after nutritional support treatment. After multiple cycles of chemotherapy, most patients would experience a decline of appetite, nausea, vomiting, as well as other chemotherapy-related adverse reactions, which further affect their energy intake. Therefore, we further analyzed the changes of BMI and SMI values during the study period, also the levels of proinflammatory cytokines before and after nutritional treatment. However, due to the short follow-up time (1 month), no significant statistical difference was found in the conclusion, but the BMI and SMI values of patients tended to increase compared with the baseline value. Based on this, we concluded that if the intervention time of nutritional therapy is further prolonged, or the drugs that can improve the appetite of patients (such as megestrol, medroxyprogesterone, etc.) (18) are added, and the gastrointestinal reaction during chemotherapy is well managed, it is possible to obtain statistically positive results that the corresponding indicators of patients are improved.

Patients with malignant tumors, especially those in the and advanced-stages, are at a very high incidence rate of developing malnutrition during treatment. The increase in the incidence of malnutrition in patients with lung cancer may be related to the excessive secretion of inflammatory cytokines caused by high tumor load, which consumes the nutrients and energy of the patients, and produces factors that inhibit the appetite of patients (19). In addition, the negative nitrogen balance caused by rapid tumor growth and the gastrointestinal reaction caused by radiotherapy and chemotherapy also affect the malnutrition of patients with decreased food intake. Malnutrition directly affects the efficacy of treatment and tolerance, reduces the quality of life, meanwhile increases the risk of in-hospital mortality and various complications in these patients (20). Early assessment of nutritional risk and targeted nutritional treatment can improve the clinical prognosis of patients. At present, there are many nutritional assessment scales for older patients, such as NRS 2002, Mini-Nutritional Assessment-Short Form (NA-SF) and Geriatric Nutritional Risk Index (GNRI). But so far there is no scale that can be used as the accepted “gold standard” for malnutrition risk assessment (20). NRS 2002 is widely used in clinical work due to its simplicity, utility and popularity. Therefore, this assessment scale is also adopted in this study as a tool of nutritional risk screening for these patients, which is of great significance for guiding rational nutritional support.

It is common knowledge that a long-term history of cigarette inhalation is a major risk factor for lung cancer. Epidemiological studies (21) have demonstrated that the incidence of sarcopenia is significant increased in olrder patients with a long-term history of cigarette intake, as well as malignant tumour patients. Cigarette intake can activate the body’s oxidative stress response, thereby degrading skeletal muscle proteins and increasing the incidence of sarcopenia (22). Based on smoking status, the patients were divided into long-term cigarette inhalation group (smoking ≥10 cigarettes/day, continuous regular smoking ≥ 10 years) and non-long-term cigarette inhalation group. Further analysis revealed that patients with lung cancer who had a long-term cigarette inhalation history had a significantly increased risk of developing sarcopenia. Oxford et al. conducted a 50-year follow-up of more than 30,000 long-term smokers and found that the pathogenesis of lung cancer, stomach cancer, oral cancer, and many other malignant tumors was related to nicotine in tobacco (22). We emphasize the importance of early smoking cessation in cancer prevention and control. Reducing cigarette intake as much as possible has important clinical value in reducing the incidence of lung cancer complicated by sarcopenia. The NRS2002 scale is widely used by nutritionists and is recommended as a quantitative tool for nutritional risk screening. Using this scale and with the help of nutritionists, we can preliminarily judge their nutritional status and determine the optimal nutritional treatment plans for them. In the present study, we observed that the incidence of malnutrition in patients with sarcopenia was significantly higher than that in those without ones. If such patients are screened for a high risk of malnutrition at the initial visit, intervention measures, such as nutritional therapy, could hopefully reduce or even reverse the occurrence of sarcopenia.

Reasonable and appropriate nutrient intake plays an important role in maintaining the normal aging rate of the body and reducing the occurrence of sarcopenia. A number of clinical studies have verified the correlation between sarcopenia and the intake of different nutrients. Hou et al. (23) conducted clinical studies showing that protein supplementation can improve the intake of muscle protein, which is not only beneficial to the synthesis of muscle fiber but also to its catabolism. This study found that 24% of patients who were not at risk for malnutrition still had sarcopenia, suggesting that sarcopenia may lead to a worse clinical outcome. Thus, even if patients are not at risk of malnutrition, as assessed through screening, there is still a need to be vigilant about sarcopenia during treatment. A low BMI score has been considered a risk factor for sarcopenia. Owing to its simple calculation method, it can be used as a preliminary screening method for patients with suspected sarcopenia. However, in this study, biochemical indicators that were more clinically evaluated, such as albumin level, triglyceride level, and lymphocyte ratio, showed no difference between the 2 groups, possibly because the ratios of serum levels of albumin, triglyceride, and lymphocytes did not change with an increase in nutrient intake or skeletal muscle mass. However, these factors can also be affected by changes in inflammatory levels caused by acute and chronic disorders such as hepatitis, tuberculosis, and infectious diseases. Therefore, they cannot be used as good indicators to assess the nutritional state of these patients.

Previous literature has reported that the mechanism of IL-6 and TNF-α induced sarcopenia may have the following aspects (24). Firstly, inflammatory cytokines can inhibit the anabolism of muscle protein, accelerate the catabolism of protein in the body, up-regulate the expression of myostatin, muscular dystrophy protein, F-box-1 Atrogin-1, etc., and promote the consumption of skeletal muscle. IL-6 can also interfere with protein synthesis and directly participate in protein breakdown, resulting in muscle reduction. In addition, high levels of IL-6 inhibit the anabolism of insulin-like growth factor I (IGF-I) in muscle tissue (25). Secondly, with the growth of age, rapid growth of tumors and modifications of body composition, cytokine products tend to increase. As human metabolism level decreases, the content of fat cells increases, and it is easier to secrete IL-6 and TNF-α, which aggravates the inflammatory response. These changes further induce the decline in skeletal muscle mass and strength, finally result in sarcopenia. Thirdly, insulin not only has the effect of lowering blood sugar, but also regulates the synthesis of target proteins that produce muscle fibers. Insulin resistance can lead to a decrease in calcium intake, which is also detrimental to muscle contraction. Therefore, elevated serum levels of these cytokines can lead to the development of insulin resistance and sarcopenia.

Recent studies have shown that inflammatory responses may also trigger mitochondrial abnormalities by disrupting the number or origin of mitochondria in cells, leading to the incidence of sarcopenia (26). A study of the association between TNF-α and sarcopenia showed that increased levels of TNF-α were related to decreased skeletal muscle mass and strength (27). Two other studies were conducted in older subjects (28): one was observed for five years in those aged 70–79, and the other was observed for 4 years in those aged 85 and older. The results showed that plasma TNF-α level could predict the decline of muscle strength. For every one standard deviation increase in TNF-α levels, muscle strength decreased by 1.2-1.3 kg. However, inflammation is only one factor in the incidence of sarcopenia, and other factors including nutritional problems and decreased hormone levels may also contribute to the decline of skeletal muscle mass and strength to varying degrees. Since malignant tumors are more common in middle-aged and older patients, sarcopenia will become an important cause of the decline in the quality of life for the older and malignant tumor patients.

In this study, the expression levels of the 2 kinds of cytokines in patients with lung cancer complicated with sarcopenia were higher than those without ones. The other two kinds of cytokines (IL-2 and IL-8) showed no significant difference between them. Through Pearson correlation analysis, it was found that both 2 cytokines were positively correlated with the incidence of sarcopenia in patients with lung cancer. Therefore, high levels of these cytokines were correlated with a higher incidence of sarcopenia. According to a study of older people in the Netherlands (29), an increase in IL-6 and C-reactive protein levels was correlated with increased muscle strength loss. *In vitro* experiments have shown that the state of synthesis and breakdown in striated muscle cells is closely correlated with TNF-α and that transcription factors play a very crucial role in this process. In experiments in mice, blocking inflammatory pathways accelerated muscle tissue regeneration (30). Oxidative stress and nitric acid products lead to decreased muscle contractility, muscle atrophy or loss of activity lead to increased TNF-α level, and the level of TNF-α can be reduced through appropriate muscle exercise (31). By observing the changes of inflammatory cytokine levels after chemotherapy intervention, we found that the difference between IL-6 and TNF-α of the 85 patients with lung cancer was gradually reduced after chemotherapy. In terms of efficacy, patients who received effective treatment (PR + CR + SD) were significantly more likely to develop disease progression (PD). Therefore, we speculate that chemotherapeutic drugs may change the secretion of inflammatory cytokines in the internal environment of these patients. However, due to the limited number of samples and observation time and the different effects of different chemotherapy regimens on sarcopenia, there may be some errors and biases.

## Limitation

Due to the limited number of samples and observation time and the different effects of different chemotherapy regimens on sarcopenia, there may be some errors and biases. To mitigate this limitation, future studies should aim to enroll a larger cohort of patients. Stratification based on chemotherapy regimens is essential to ensure balanced subgroup comparisons. Prospective study designs are needed to systematically evaluate the effects of different chemotherapy regimens and multicycle chemotherapy interventions on cytokine levels in both patient groups. Further investigation is required to fully elucidate the potential association between lung cancer with comorbid sarcopenia and treatment outcomes. Mechanistic studies should prioritize exploring how chemotherapy regulates inflammatory cytokine levels in this patient population.

## Conclusions

In summary, developing good living habits, quitting smoking, performing proper exercise, conducting regular nutritional risk assessment for patients with lung cancer and ensuring early detection and avoidance of malnutrition risk can help reduce the occurrence of sarcopenia. Elevated levels of partial inflammatory cytokines are related to the occurrence of lung cancer complicated with sarcopenia. After chemotherapy, the survival time of patients with lung cancer and sarcopenia can be increased to a certain extent, providing a new prospective for improving the overall clinical prognosis of patients with lung cancer and early detection of high-risk sarcopenia cases.

## Data Availability

The datasets presented in this study can be found in online repositories. The names of the repository/repositories and accession number(s) can be found in the article/supplementary material.
